# Asymmetric dimethylarginine and all-cause mortality: a systematic review and meta-analysis

**DOI:** 10.1038/srep44692

**Published:** 2017-03-15

**Authors:** Shaoli Zhou, Qianqian Zhu, Xiang Li, Chaojin Chen, Jiping Liu, Yuping Ye, Ying Ruan, Ziqing Hei

**Affiliations:** 1 Department of Anesthesiology, The Third Affiliated Hospital of Sun Yat-Sen University, Guangzhou City, People’s Republic of China; 2 Foshan Women and Children’s Healthcare Hospital, Foshan City, People’s Republic of China; 3 Department of Thyroid and Breast Surgery, The Third Affiliated Hospital of Sun Yat-Sen University, Guangzhou City, People’s Republic of China

## Abstract

Asymmetrical dimethylarginine (ADMA), an endogenous inhibitor of nitric oxide synthase (NOS), impairs the beneficial effect of NO. The predictive value of ADMA for all-cause mortality remains controversial, though it is important in the development of cardiovascular disease (CVD) and progression to dialysis in renal disease. This systematic review and meta-analysis was conducted to investigate the association between circulating ADMA and all-cause mortality. Studies with data pertinent to the association between circulating ADMA and all-cause mortality were reviewed and OR, HR or RR with 95% CI derived from multivariate Cox’s proportional-hazards analysis were extracted. A total of 34 studies reporting 39137 participants were included in final analysis. The results demonstrated that circulating ADMA was independently associated with all-cause mortality (RR = 1.27, 95% CI: 1.20–1.34). The association was still statistically significant in patients with pre-existing renal disease (RR = 1.30, 95% CI: 1.19–1.43) and pre-existing CVD (RR = 1.26, 95% CI: 1.16–1.37). In those without pre-existing renal or CVD, ADMA also predicted all-cause mortality (RR = 1.31, 95% CI: 1.13–1.53). The present study suggests a positive association of circulating ADMA with all-cause mortality. Further studies are needed to investigate the effects of interventions on ADMA, and the value of ADMA as a biomarker.

Asymmetrical dimethylarginine (ADMA), an endogenous inhibitor of nitric oxide synthase (NOS), inhibits nitric oxide (NO) biosynthesis^1^. Therefore, increase in circulating ADMA may impair the beneficial effects of NO on endothelial function, vasodilatation, and arterial stiffness^2^. Elevation of ADMA has been observed to be associated with hypertension, diabetes, hypertriglyceridemia, and hyperhomocysteinemia. These are risk factors for cardiovascular and cerebrovascular diseases^3^,^4^,^5^. Furthermore, studies have shown that a high circulating ADMA concentration is associated with a higher incidence of cardiovascular disease (CVD) and worse cerebrovascular disease outcomes^6^,^7^.

Owing to its involvement in CVD, circulating ADMA as a predictor of risk for mortality in patients with pre-existing CVD has been investigated. The prognostic value of ADMA has been well documented in patients with dilated cardiomyopathy, diabetes mellitus, and acute ischemic stroke^8^,^9^. Additionally, in patients with chronic kidney disease, ADMA has also been shown to be an important predictor of progression to end-stage renal disease (ESRD) and all-cause mortality^10^. Circulating ADMA is also associated with all-cause mortality in patients with diabetes mellitus^11^.

Even in a healthy population, high levels of circulating ADMA may be associated with higher rates of all-cause death^12^. However, the predictive value of ADMA for all-cause mortality remains controversial. Some researchers have reported that ADMA is not an independent predictor of all-cause mortality after multivariate adjustment^13^,^14^. The complicated interpretation of the predictive value of ADMA might be attributed to the limited number of participants involved in each individual study and reduced mortality. Therefore, it is important to clarify the relationship between ADMA and survival for potential interventions. To investigate the association between circulating ADMA and all-cause mortality, we conducted a systematic review and meta-analysis.

## Methods

### Literature search

We searched the following databases: Cochrane Library, PubMed, Web of Knowledge, and Elsevier (ScienceDirect OnLine) to retrieve literature investigating the association between circulating ADMA and all-cause mortality. The terms “ADMA or asymmetrical dimethylarginine” and “mortality or survival or outcomes or prognosis or prognostic” were used as search terms. Eligible trials were identified up to June 1, 2016 through electronic searches. Hand searches of the references of the identified trials were also conducted. The meta-analysis was conducted in accordance with the PRISMA guidelines.

### Inclusion and exclusion criteria

Studies were considered for inclusion if they met the following criteria: (i) written in English, (ii) reported the all-cause mortality of participants, (iii) investigated the relationship between circulating ADMA with all-cause mortality, (iv) reported hazard ratio (HR), relative risk (RR), or odds ratio (OR) with 95% confidence intervals (95% CI) for ADMA associated with all-cause mortality, and (v) used a multivariate Cox’s proportional-hazards model to analyze the HR, RR, or OR value. Studies were excluded on the basis of the following criteria: (i) not written in English, (ii) used univariate analyses alone for the correlation of plasma ADMA with all-cause mortality, and (iii) the full-text article was not available.

### Data extraction

The data were extracted independently by two reviewers (Qianqian Zhu and Xiang Li) and validated by a third reviewer (Shaoli Zhou). The following information was extracted: primary author, year of publication, geographical location, number of participants, sex ratio, and baseline average age (mean, median, or range).

### Statistical analysis

Meta-analysis was performed in Review Manager 5 (The Cochrane Collaboration, Oxford, UK). The pooled effect of ADMA as a predictor of all-cause mortality was calculated as RR with 95% CI. A chi-square test was used to assess the heterogeneity. I^2^ value <25% was defined as no heterogeneity and a random-effects model was used when heterogeneity existed among the studies analyzed. A Begg and Egger test was used to test the publication bias and analyses were performed by using Stata 12.1 (Stata Corp., College Station, TX). Differences were considered significant when the two-tailed p values were <0.05.

## Results

### Search

**Figure 1:**
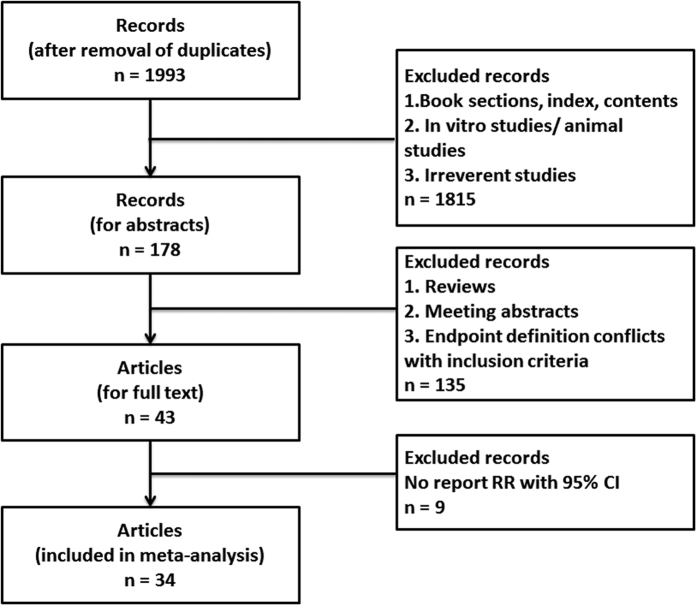
The flow chart of inclusion and exclusion.

The search strategy yielded a total of 1993 non-duplicated entries. After screening the titles, the type of entries, and the abstracts, 43 articles were chosen for full review. We identified 34 eligible studies for the final analysis. The inclusion and exclusion process of the studies is shown in Fig. 1.

### Characteristics of the included studies

A total of 34 studies reporting a total of 39137 participants were included in the final analysis (Table 1)^7^,^9^,^11^,^13^,^14^,^15^,^16^,^17^,^18^,^19^,^20^,^21^,^22^,^23^,^24^,^25^,^26^,^27^,^28^,^29^,^30^,^31^,^32^,^33^,^34^,^35^,^36^,^37^,^38^,^39^,^40^,^41^,^42^,^43^. Eleven studies were based on 9319 participants with pre-existing renal diseases including 2533 renal transplant recipients^7^,^15^,^19^,^21^,^22^,^25^,^26^,^28^,^38^,^39^,^43^. Nineteen studies included participants at high cardiovascular events risk^9^,^11^,^13^,^17^,^18^,^20^,^26^,^27^,^29^,^30^,^31^,^32^,^35^,^36^,^37^,^40^,^41^,^42^,^44^. Of these 19 studies, 14 studies involved 15584 participants with pre-existing cardiovascular disease^9^,^13^,^18^,^26^,^27^,^29^,^30^,^32^,^35^,^36^,^37^,^40^,^41^,^42^.

### Circulating ADMA concentration and all-cause mortality for all participants

**Figure 2:**
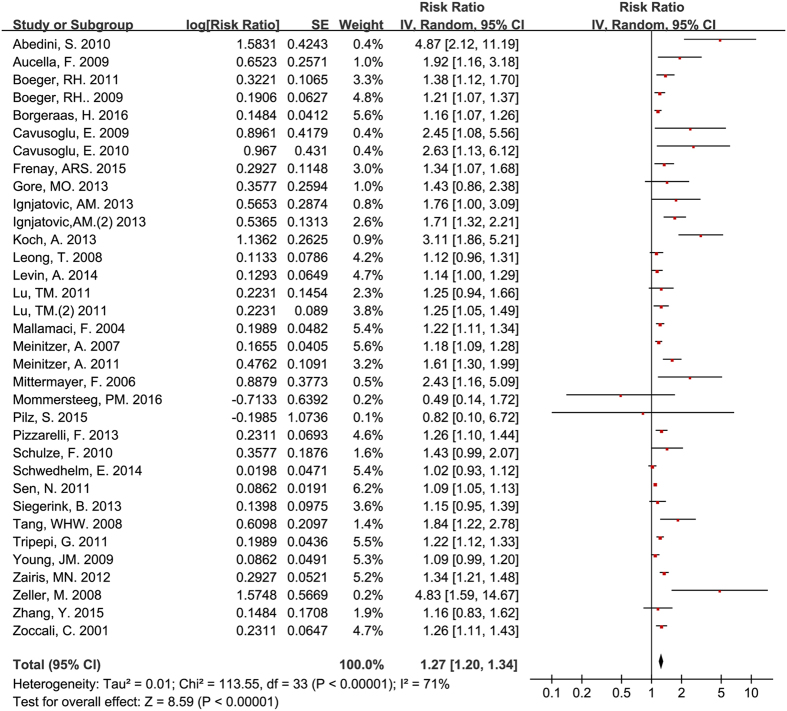
Circulating ADMA concentration and all-cause mortality for all participants.

All of the 34 studies included in the meta-analysis provided a multivariate HR, RR, or OR with a 95% CI for the pooled predictive effect of ADMA for all-cause mortality. Our results demonstrated that ADMA was independently associated with all-cause mortality (RR = 1.27, 95% CI: 1.20–1.34, Fig. 2). Moderate heterogeneity (I^2^ = 71%) existed for these studies. After excluding the four studies^11^,^21^,^26^,^29^ with fewer participants that might have come from the same study group used in four other studies^9^,^22^,^27^,^30^, circulating ADMA was still associated with all-cause mortality (RR = 1.25, 95% CI: 1.18–1.31), and the level of between-study heterogeneity was moderate (I^2^ = 71%).

### Circulating ADMA concentration and all-cause mortality for participants with pre-existing renal diseases

**Figure 3:**
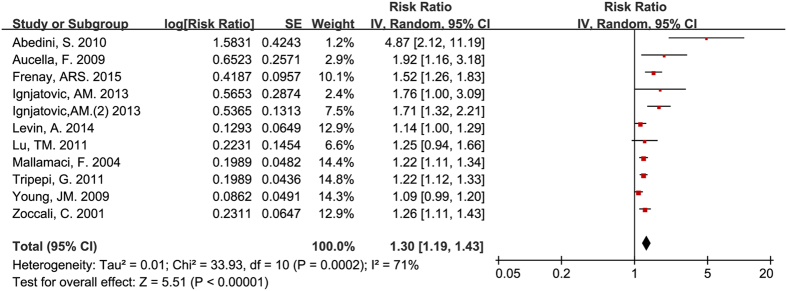
Circulating ADMA concentration and all-cause mortality for participants with pre-existing renal diseases.

After analyzing the data from 11 studies based on participants with pre-existing renal diseases^7^,^15^,^19^,^21^,^22^,^25^,^26^,^28^,^38^,^39^,^43^, we found that circulating ADMA still showed a predictive value for mortality (RR = 1.30, 95% CI: 1.19–1.43, Fig. 3). The value remaining after excluding one of the two studies that used patients from the same study group was almost identical (RR = 1.27, 95% CI: 1.16–1.39)^21^,^22^. However, the heterogeneity in both the 11 (I^2^ = 71%) and the 10 studies was significant (I^2^ = 67%).

### Circulating ADMA concentration and all-cause mortality for participants with pre-existing cardiovascular disease

**Figure 4:**
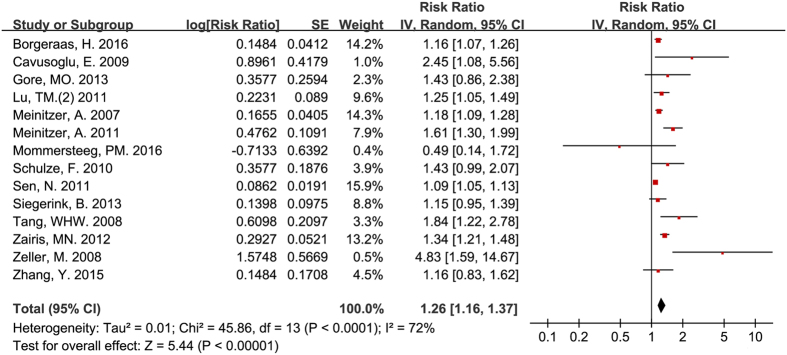
Circulating ADMA concentration and all-cause mortality for participants with pre-existing cardiovascular disease.

Fourteen studies included patients with pre-existing cardiovascular disease^9^,^13^,^18^,^26^,^27^,^29^,^30^,^32^,^35^,^36^,^37^,^40^,^41^,^42^. Circulating ADMA was also an independent predictor for all-cause mortality for participants with pre-existing cardiovascular disease (RR = 1.26, 95% CI: 1.16–1.37, Fig. 4). There was moderate heterogeneity among studies (I^2^ = 72%).

After excluding one of the two studies using patients from the same study group, circulating ADMA was still associated with all-cause mortality (RR = 1.23, 95% CI: 1.13–1.33)^29^,^30^.

### Circulating ADMA concentration and all-cause mortality for participants without pre-existing renal diseases or CVD

**Figure 5:**
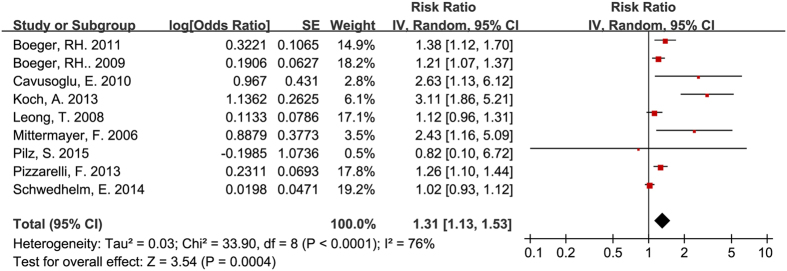
Circulating ADMA concentration and all-cause mortality for participants without pre-existing renal diseases or CVD.

Consistent with the results of studies involving participants with CVD or renal disease, circulating ADMA was independently associated with all-cause mortality for participants without CVD or renal disease (RR = 1.31, 95% CI: 1.13–1.53, Fig. 5). The heterogeneity among studies was moderate (I^2^ = 76%).

### Circulating ADMA concentration and cardiac events for all participants

**Figure 6:**
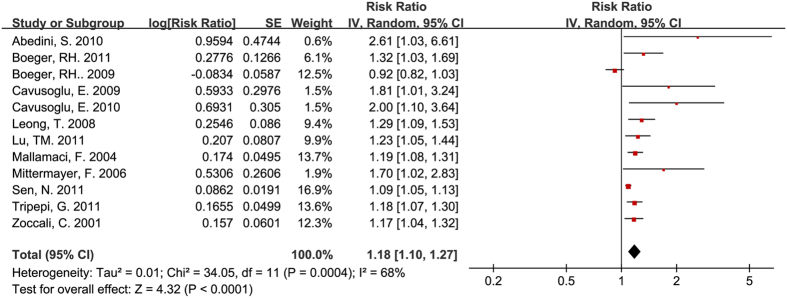
Circulating ADMA concentration and major cardiovascular events.

**Figure 7:**
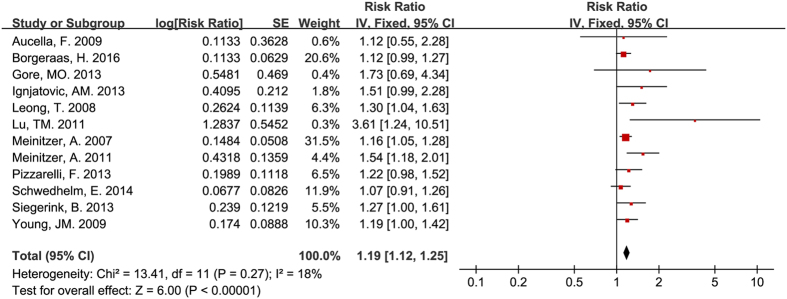
Circulating ADMA concentration and cardiovascular death.

Statistically significant associations existed between circulating ADMA and major cardiovascular events (RR = 1.18, 95% CI: 1.10–1.27, Fig. 6) and cardiovascular death (RR = 1.19, 95% CI: 1.12–1.25, Fig. 7).

### Publication bias

**Figure 8:**
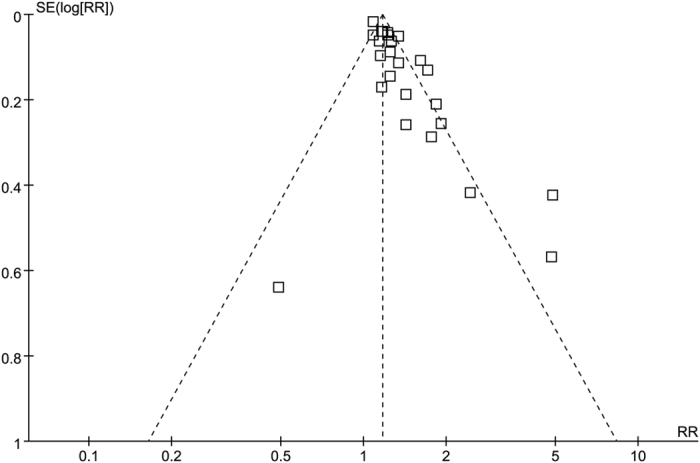
Funnel plot for all publication.

The funnel plot showed that there might be publication bias (Fig. 8). A Begg and Egger test was then used to test the publication bias for all studies and the studies enrolling participants with pre-existing diseases (p < 0.001 for both).

There was no significant publication bias for the studies that enrolled participants with pre-existing renal diseases (p = 0.306). However, publication bias was observed for studies enrolling participants with pre-existing cardiovascular diseases (p = 0.017).

## Discussion

We performed a systematic review and meta-analysis to investigate the association between circulating ADMA and all-cause mortality. Our analysis showed that high circulating ADMA was independently associated with all-cause mortality, and the association remained in patients with or without pre-existing renal disease and pre-existing CVD. In addition, high circulating ADMA was also associated with major cardiovascular events or cardiovascular death.

An elevation of circulating ADMA in patients with CVD and pre-existing renal disease has been reported^9^,^18^. A previous study reported that high ADMA was associated with CVD events, consistent with the results of the present subgroup analyses that high circulating ADMA was associated with both major cardiovascular events and cardiovascular death^24^. Additionally, previous studies also indicated that a high circulating ADMA concentration was inversely related to glomerular filtration rate and positively correlated with progression to dialysis^10^,^26^. Furthermore, ADMA could predict mortality in patients with CVD or chronic renal disease^18^,^21^.The predictive value of ADMA for CVD outcomes remained significant even in participants without pre-existing CVD or kidney disease at baseline^6^.

However, studies investigating the relationship between ADMA and all-cause mortality reported inconsistent results. Although some studies reported that ADMA was significantly associated with all-cause mortality^11^,^16^,^18^, other studies did not find that ADMA was associated with all-cause mortality^32^,^33^,^42^. The different results might be because of the limited number of participants involved in each single-center study in addition to the complicated role of ADMA *in vivo*.

Our present study included data from 34 studies with more than 35000 participants and our results suggest that increased levels of circulating ADMA are an independent predictor for all-cause mortality after multivariate Cox’s proportional-hazards model adjustment. The association was similar in patients with or without pre-existing chronic renal disease and pre-existing CVD.

The main results of our present study are consistent with a recent published meta-analysis by Schlesinger and colleagues that showed that ADMA was an independent risk marker for all-cause mortality and CVD^45^. However, we searched more databases than they did and included as many studies as possible. We also tested for publication bias.

Furthermore, Review Manager was used to carry out the meta-analysis evaluating the weight of each study to determine which studies influenced the final results more than the others did. In our present study, after excluding the studies ranked in the top three weights, significance remained in every meta-analysis. For example, after excluding the top three weighted studies^18^,^30^,^46^, ADMA was still independently associated with all-cause mortality (RR = 1.31, 95% CI: 1.23–1.40). In addition, although the studies included heterogeneous populations with different conditions at baseline or different interventions, we only included the HR, RR, or OR value from multivariate Cox’s proportional-hazards model analyses, which reduced the bias.

For both CVD and chronic renal disease, the relationship between ADMA and outcomes might involve endothelial dysfunction. The endothelial dysfunction might be because of disturbed NO regulation, which would lead to impaired biological activity of NO^2^,^47^. NO deficiency is attributed to two possible causes, substrate (L-arginine) limitations and increased levels of circulating endogenous inhibitors of NOS, particularly ADMA. Therefore, the elevation of circulating ADMA might be partly reflecting the imbalance of arginine and ADMA ratio that affects NO production and has been shown to be related to changes in microcirculation of major organs and increased hospital mortality^48^,^49^. In addition to its important role in endothelial regulation, NO is also involved in vascular smooth muscle cell growth, platelet aggregation, and leukocyte adhesion, which play important roles in microcirculation^50^. The reduction of NO production in glomerular endothelia might cause vascular damage and enhance endothelial adhesion of leukocytes and platelets, which would subsequently increase the mortality of patients with renal disease^21^.

In addition to its role in endothelial dysfunction and microcirculation, various studies have reported that ADMA is consistently associated with biomarkers of inflammation in chronic conditions including diabetes, renal disease, and hypertension^38^,^51^,^52^. ADMA can induce TNF-α and IL-8 production via oxidative stress due to generation of reactive oxygen species/NF-κB-dependent pathway *in vitro*^53^. Therefore, circulating ADMA might be a potential pro-inflammatory factor in addition to inhibiting NOS.

Furthermore, the prognostic role of ADMA in all-cause mortality might involve additional underlying mechanisms. ADMA belongs to a family of amino acid methylation derivatives including N-mono-methylarginine (MMA), the immediate precursor to ADMA, and symmetric dimethylarginine (SDMA), a stereoisomer of ADMA^54^. Low plasma MMA is the most potent NOS inhibitor and has been found to be inversely related to cardiovascular disease outcomes^55^. Unlike ADMA, SDMA lacks NOS inhibitory activity. However, SDMA is a weak inhibitor of arginine transporters^54^. Studies have also reported that SDMA is associated with an increased prevalence of major adverse cardiac events, renal dysfunction, and all-cause mortality^55^,^56^. These derivatives mentioned above also show predictive value for mortality in patients with pre-exiting CVD or renal disease^55^,^56^. Furthermore, some findings suggest that ADMA may directly promote vascular disease^57^. Therefore, these findings raised the possibility that the underlying mechanisms of ADMA in predicting all-cause mortality might be partly independent of NOS inhibition and NO production.

Various studies have explored the predictive value of some biomarkers in patients with cardiac failure and those undergoing hemodialysis^58^,^59^,^60^. However, none have had satisfactory high sensitivity and specificity. Since elevated plasma ADMA concentrations independently predict CVD and renal disease outcomes, ADMA may have potential utility as a clinical biomarker. Ideal biomarkers should also exhibit pharmacologic responses to a therapeutic intervention. Some drugs such as statins and angiotensin converting enzyme inhibitors and angiotensin receptor blockers can affect ADMA^61^. A meta-analysis reported a significant reduction in plasma ADMA concentrations following statin therapy^62^. However, the cut-off value of ADMA in prediction, whether the absolute increase in ADMA is clinically relevant, and whether earlier or more aggressive intervention can improve clinical outcomes remains unclear. Therefore, studies exploring the above-mentioned unclear problems are needed in the future.

Several limitations of our present study should be considered. First, not all of the studies were prospective, which might lead to biases. Second, the substantial heterogeneity of the included studies might decrease the power of the results. Third, we only included published studies written in English. This might lead to publication bias. However, the large number of participants included in the present meta-analysis provided sufficient data for calculation of the pooled effect of ADMA on all-cause mortality. Furthermore, we only used the OR, RR, or HR with 95% CI from multivariate Cox’s proportional-hazards analysis to calculate the pooled predictive value of ADMA on mortality, which should reduce any potential bias.

In summary, the present study suggests a positive association of circulating ADMA with all-cause mortality. Further studies are needed to investigate the associations in the general population, the effects of interventions on ADMA, and the value of ADMA as a biomarker, especially in patients with CVD and those with renal diseases.

## Additional Information

**How to cite this article**: Zhou, S. *et al*. Asymmetric dimethylarginine and all-cause mortality: a systematic review and meta-analysis. *Sci. Rep.* **7**, 44692; doi: 10.1038/srep44692 (2017).

**Publisher's note:** Springer Nature remains neutral with regard to jurisdictional claims in published maps and institutional affiliations.

## Figures and Tables

**Table 1:** Characteristics of studies included in meta-analysis.

Author, year	Country	Baseline characteristics	No. of participants	Mean age (y)	Male (%)	Follow-up time
Abedini, Meinitzer *et al*.^7^	Norway	renal transplant recipients	2102	40.7	N/A	6 y (max)
Aucella, Maas *et al*.^15^	Puglia	haemodialysis and dialysis	288	58	57%	56 m (mean)
Boeger, Endres *et al*. 2011	Germany	Framingham Offspring	2447	73.0	43.7%	5 y (max)
Boger, Sullivan *et al*. 2009	UAS	Framingham Off spring	3320	59	45.7%	10.9 y (mean)
Borgeraas, Hertel *et al*.^18^	Norway	suspected stable angina pectoris undergoing coronary angiography	4122	62	72%	4.7 y (mean)
Cavusoglu, Ruwende *et al*.^9^	USA	acute coronary syndrome referred for coronary angiography	182	64.8	100%	2 y (max)
Cavusoglu, Ruwende *et al*.^11^	USA	diabetes mellitus referred for coronary angiography	162	65.9	100%	2 y (max)
Frenay, van den Berg *et al*.^19^	Netherlands	Renal transplant recipients	686	53.0	57%	3.1y (mean)
Gore, Luneburg *et al*.^20^	UK	Dallas Heart Study	3523	43	44%	7.4 y (mean)
Ignjatovic, Cvetkovic *et al*.^21^,^22^	Serbia	dialysis patients	153	58	68.6%	3y (max)
Ignjatovic, Cvetkovic *et al*.^21^,^22^		hemodialysis	162	N/A	N/A	14 m (N/A)
Koch, Weiskirchen *et al*.^23^	Germany	critically ill patients	255	63(median)	58.4%	3y (max)
Leong, Zylberstein *et al*.^24^	Norway	women in the Population Study (helath)	880	N/A	0%	24 y (max)
Levin, Rigatto *et al*.^25^	Canadian	chronic kidney disease	2544	68.1	63%	1y (mean)
Lu, Chung *et al*.^26^,^27^	Taiwan	stage 3 to 4 CKD	298	73	85.9%	2.7y (mean)
Lu, Chung *et al*.^26^,^27^	Taiwan	referred for coronary angiography	997	66.9	79%	2.4 y (mean)
Mallamaci, Tripepi *et al*.^28^	Italy	end-stage renal disease	224	54.9	60	42.3 (mean)
Meinitzer, Kielstein *et al*.^29^	Germany	referred for coronary angiography	3229	N/A	N/A	7.7 y (mean)
Meinitzer, Seelhorst *et al*.^30^	Germany	angiographic coronary artery disease	3238	62.7	69.7%	5.45 y (mean)
Mittermayer, Krzyzanowska *et al*.^31^	Austria	advanced peripheral artery disease	496	70	56.3%	19 m (mean)
Mommersteeg, Schoemaker *et al*.^32^	Netherlands	heart failure	104	65.7	72%	6.1 y (mean)
Pilz, Putz-Bankuti *et al*.^33^	Austria	chronic liver disease	94	59	69.1%	3.5 y (mean)
Pizzarelli, Maas *et al*.^34^	Italy	elderly	1025	75	44%	110 m (mean)
Schulze, Carter *et al*.^13^	UK	acute ischemic stroke	394	69.9	53.5%	7.4 y (mean)
Schwedhelm, Wallaschofski *et al*.^14^	Germany	study of Health in Pomerania	3952	51	49%	10.1 y (mean)
Sen, Ozlu *et al*.^35^,^46^	Turkey	acute myocardial infarction patients	168	57.4	70%	1 y (max)
Siegerink, Maas *et al*.^36^	Germany	stable coronary heart disease	1148	58.7	84.6%	8.1 y (mean)
Tang, Tong *et al*.^37^	Cleveland	chronic systolic heart failure	132	57.8	77%	33 m (mean)
Tripepi, Mattace Raso *et al*.^38^	Germany	hemodialysis patients	225	60	55%	13 y (max)
Young, Terrin *et al*.^39^	USA	stages 3 to 4 chronic kidney disease	820	52	60%	9.5 y (mean)
Zairis, Patsourakos *et al*.^40^	Greece	chronic heart failure	651	73	64.1%	1 y (max)
Zeller, Korandji *et al*.^41^	France	acute myocardial infarction	249	68.7	78%	1 y (max)
Zhang, Blasco-Colmenares *et al*.^42^	USA	heart failure (PROSE-ICD)	402	60.1	73.6%	5.5y (mean)
		heart failure (GRADE)	240	62.5	77.1%	3.7y (mean)
Zoccali, Bode-Boger *et al*.^43^	Germany	hemodialysis patients	225	59.9	54.7%	33.4 m (mean)
